# Supplementary material for: Magnetic voluntary head-fixation in transgenic rats enables lifespan imaging of hippocampal neurons
Source: Nat Commun. 2024 May 16;15:4154. doi: 10.1038/s41467-024-48505-9 (PMC11099169; doi:10.1038/s41467-024-48505-9)
Supplement: Supplementary file 1 — Supplementary Information [file 41467_2024_48505_MOESM1_ESM.pdf]

Supplementary figures

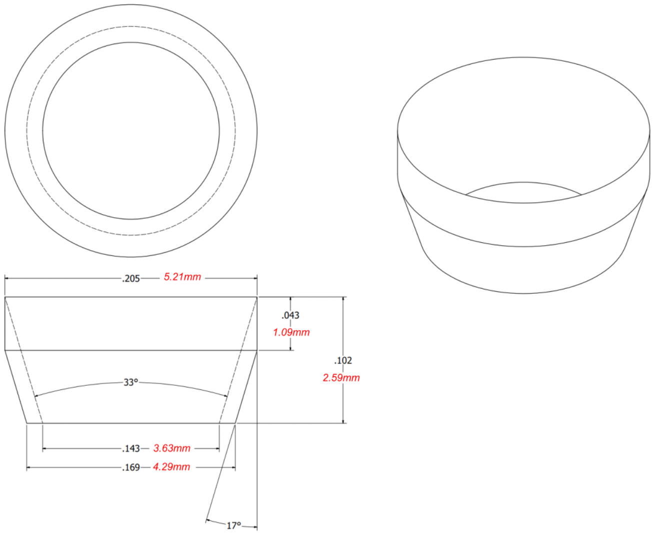

Figure S1 – Technical drawing and dimensions of the conical cannula.
Black dimensions are in inches, and red dimensions are in mm.

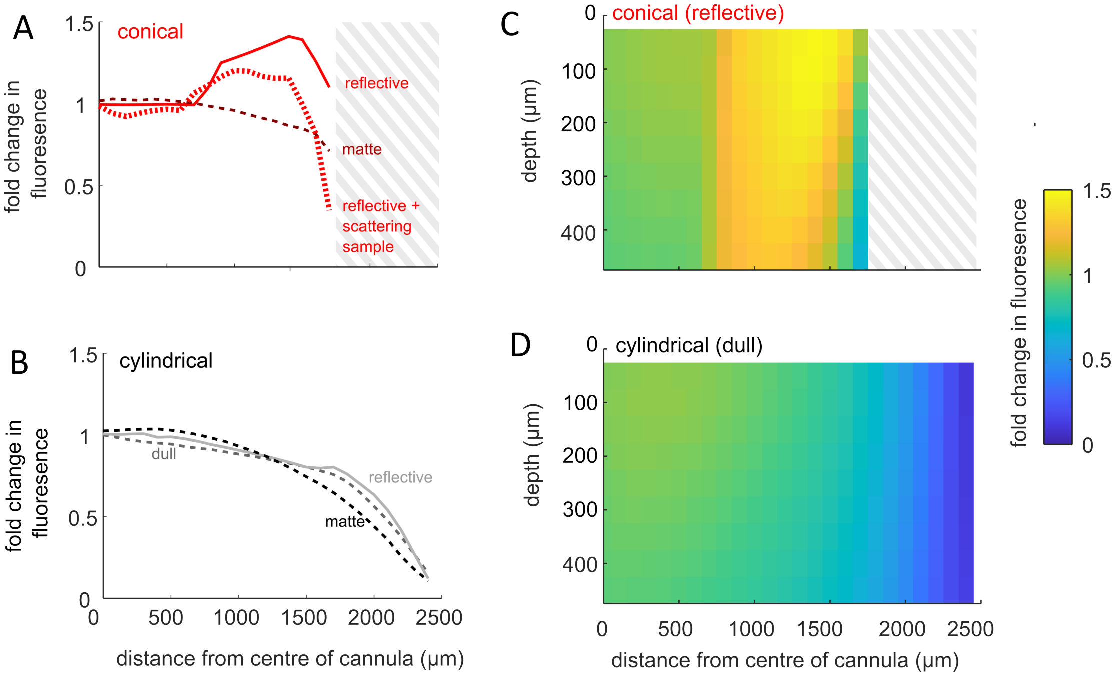

Figure S2 – Bench tests of the various cannula configurations

A and **B** show the fold change in fluorescence as a function of the distance from the cannula center, measurements taken at a depth of 150µm. **A** For the conical cannula, showing the same cannula with a reflective and matte finish. Also showing the reflective cannula with and a scattering sample. **B** For the cylindrical cannula we show three finishes, reflective, dull (unfinished) and matte. **C** and **D** show the fold change in fluorescence as a function of the imaging depth. Each row is normalized by the level of fluorescence in the center of the cannula in order to isolate from the effect of decreased excitation at different depths.

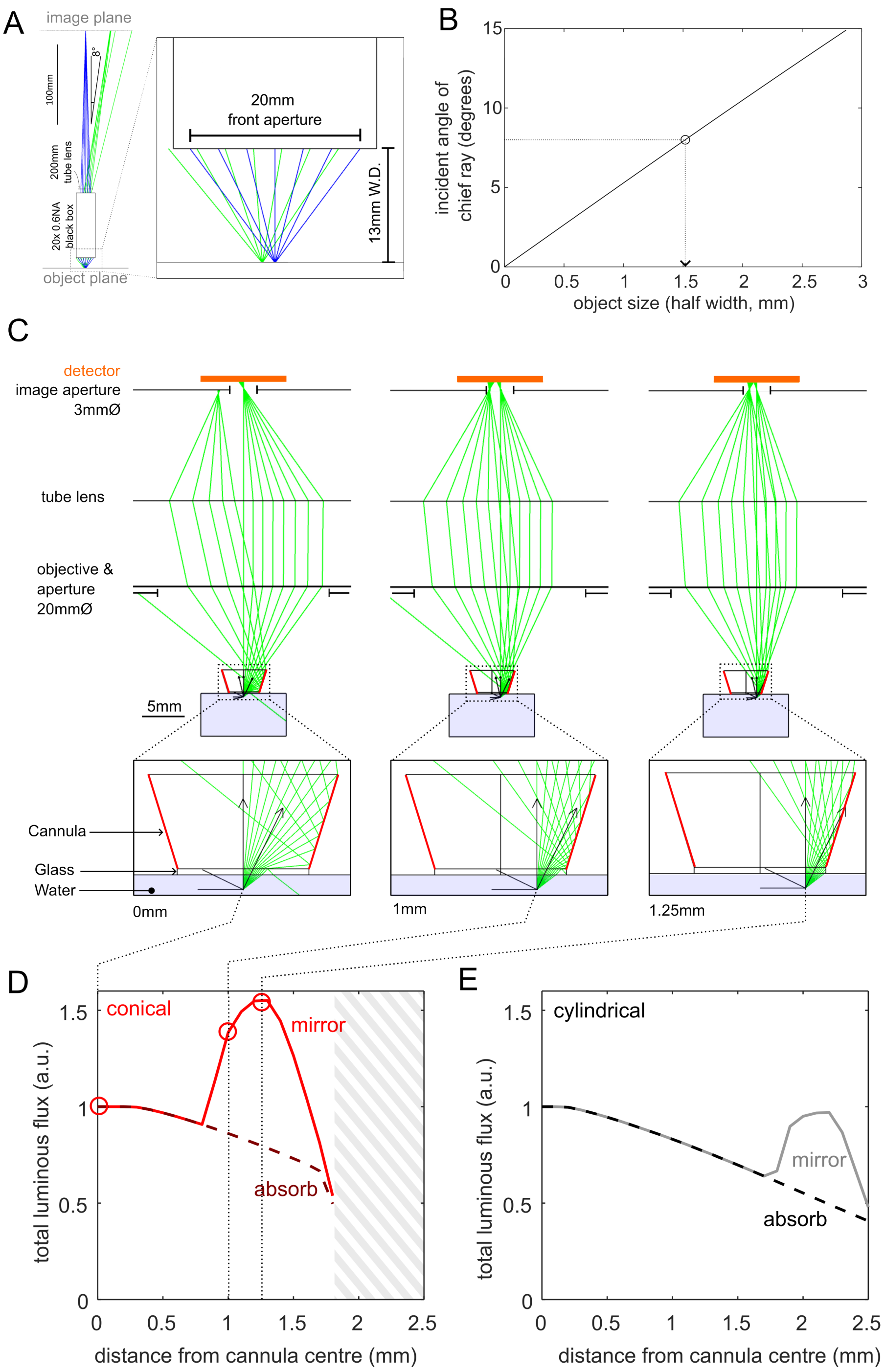

Figure – S3 Ray-tracing of cannula collection.

A Sequential ray tracing configuration used to determine the image aperture diameter, which describes the spatio-angular acceptance of the objective and collection optics. Blackbox model of objective is used with a tube lens to create an image. **B** The incident angle of the chief ray as a function of the object size provides an object field of view size that corresponds to a back-aperture ray angle of 8 degrees, the 50% transmission angle of the collection system. **C** The configuration for non-sequential ray tracing analysis. Three views are shown for separate radial distances of the fluorescent source from the cannula center.

Only rays in a fan from 0 degrees to past the internal reflection critical angle for glass-air are shown in green for clarity, although rays used for calculation are launched at all angles from the point source. **D** Plot of the total luminous flux as a function of the distance of the fluorescent source from the cannula center, for a mirror vs absorbing cannula. Hatched area corresponds to invalid region since the conical cannula has a smaller bottom diameter. **E** as D but for the conventional cylindrical cannula. The obstruction of the excitation beam as a function of lateral position can be derived from the ray-tracing data, because of the optical principle of reversibility, the total luminous flux in the absorb condition is equivalent to the obstruction.

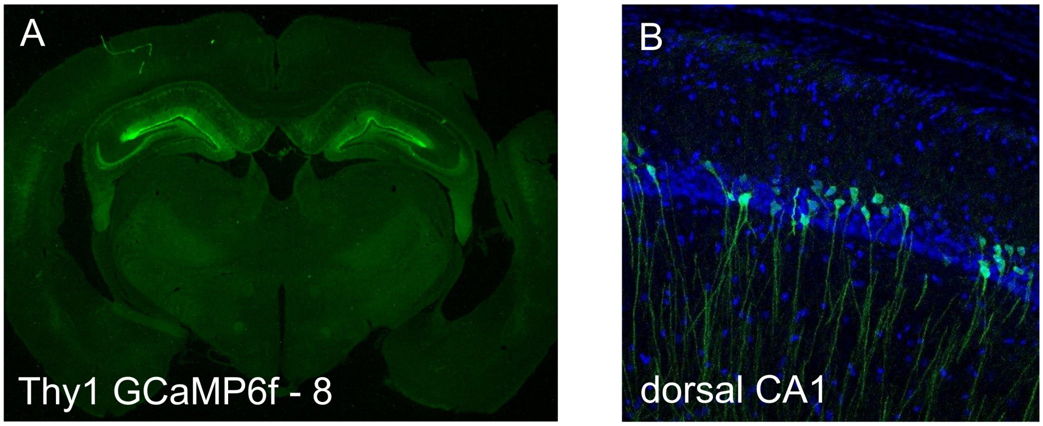

Fig S4 - Transgenic expression of GCaMP6f

A – Epifluorescence microscopy image of native GCaMP6f expression (green) in a coronal section from a Thy1-Gcamp6f- 8 rat. **B** Confocal microscopy image of the of native GCaMP6f expression (green) and DAPI (blue) of the CA1 layer of the dorsal hippocampus.

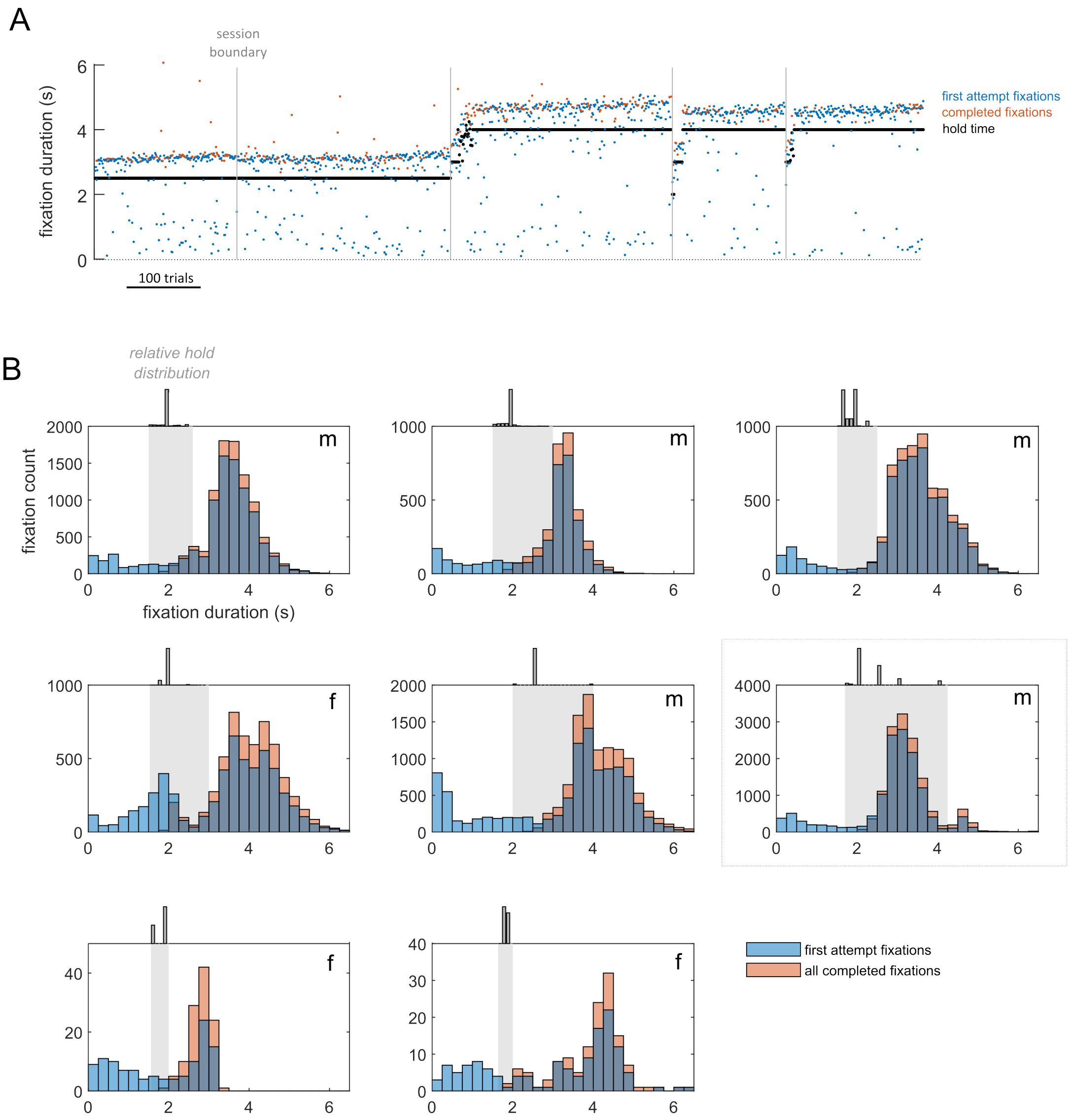

Figure S5 - Fixation durations

A – Example fixation durations for 5 sessions for a single animal (dotted box in B). Two initial sessions require a hold of 2.5s, and in the 3rd session the animal is quickly trained to perform 4s holds. Blue dots show the first attempt fixations for each trial, and the red dots show the duration of the completed fixations that were not achieved on the first attempt (animals have to complete the required duration to proceed to the next trial). **B -** Distribution of the fixation durations for 8/9 animals (remaining animal was trained on longer durations and the full distribution is shown in Fig. S6) for all sessions following initial training. Blue bars are distribution of fixation times for the first fixation attempt per trial, (which includes fixations which are too short). Red is the distribution of all completed fixations, one for each trial. The hold duration range (determined by the experiment) is given by the grey box area, and the histogram above shows the relative distribution of hold durations across trials. The most common hold duration is 2s, but shorter holds were used for example when animals were coming back on task after a break. Longer duration holds (up to 4s) were also used for two animals. The animal sex is indicated in the top right corner. The total proportion of trials that were completed (i.e. made the minimum prescribed hold duration) on the initial fixation for a given trial was 0.78,0.82,0.84,0.89,0.72,0.60,0.73,0.75 for each animal.

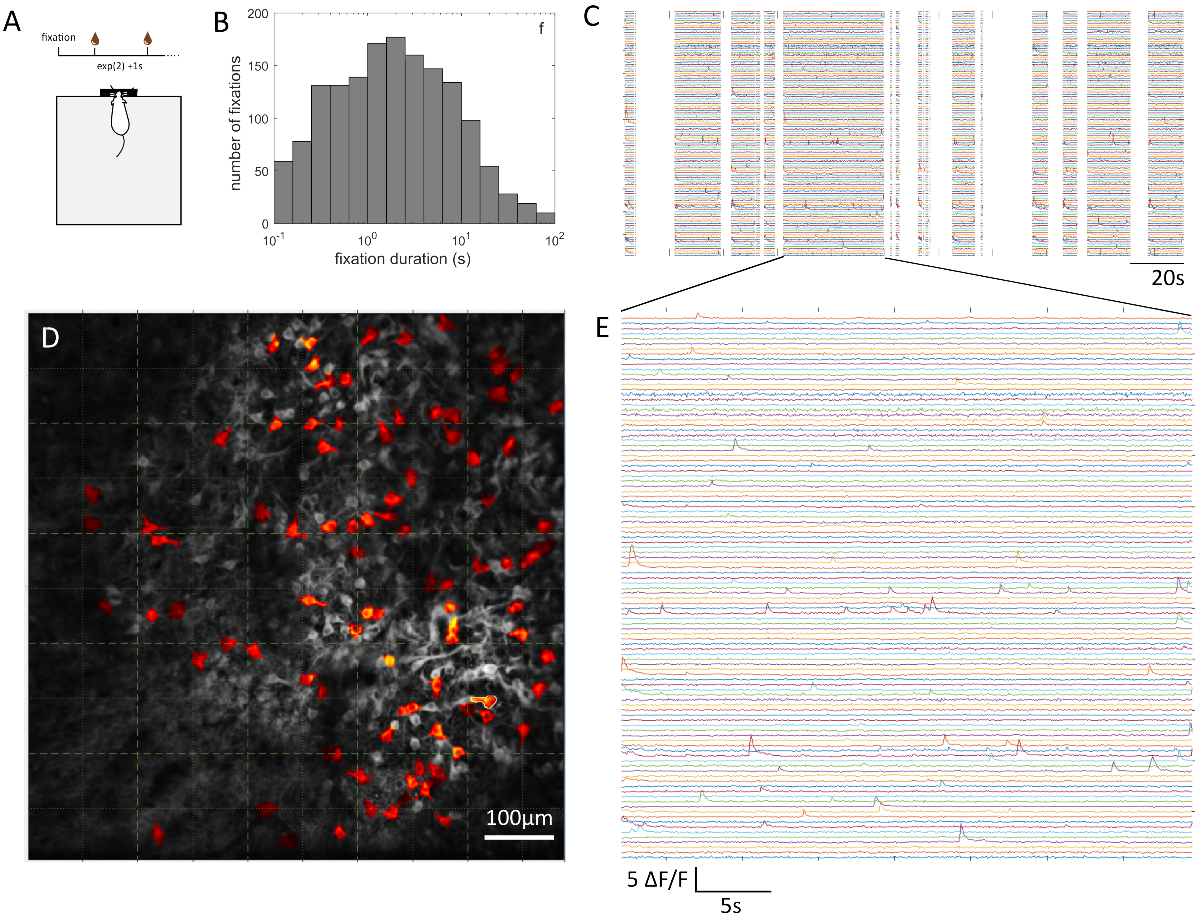

Figure S6 – Long fixation imaging.
A – Showing the long fixation experiment in which one animal is required to hold fixation for as an indefinite period, during which time it gets random rewards with an interval given by a random draw from an exponential with a mean of 2s plus 1s. **B –** Distribution of all fixation times for the one animal trained on indefinite holds. This animal was female. Showing data for 9 sessions, the mean was 4.7s and the maximum hold being 70s. Note there is no incentive for continued long holds since the average reward rate is constant **C –** Calcium fluorescence time courses of active cells during fixation. **D –** Field of view showing average fluorescence and highlighted active cells. **E –** Zoom of calcium activity for a single fixation of 37s.

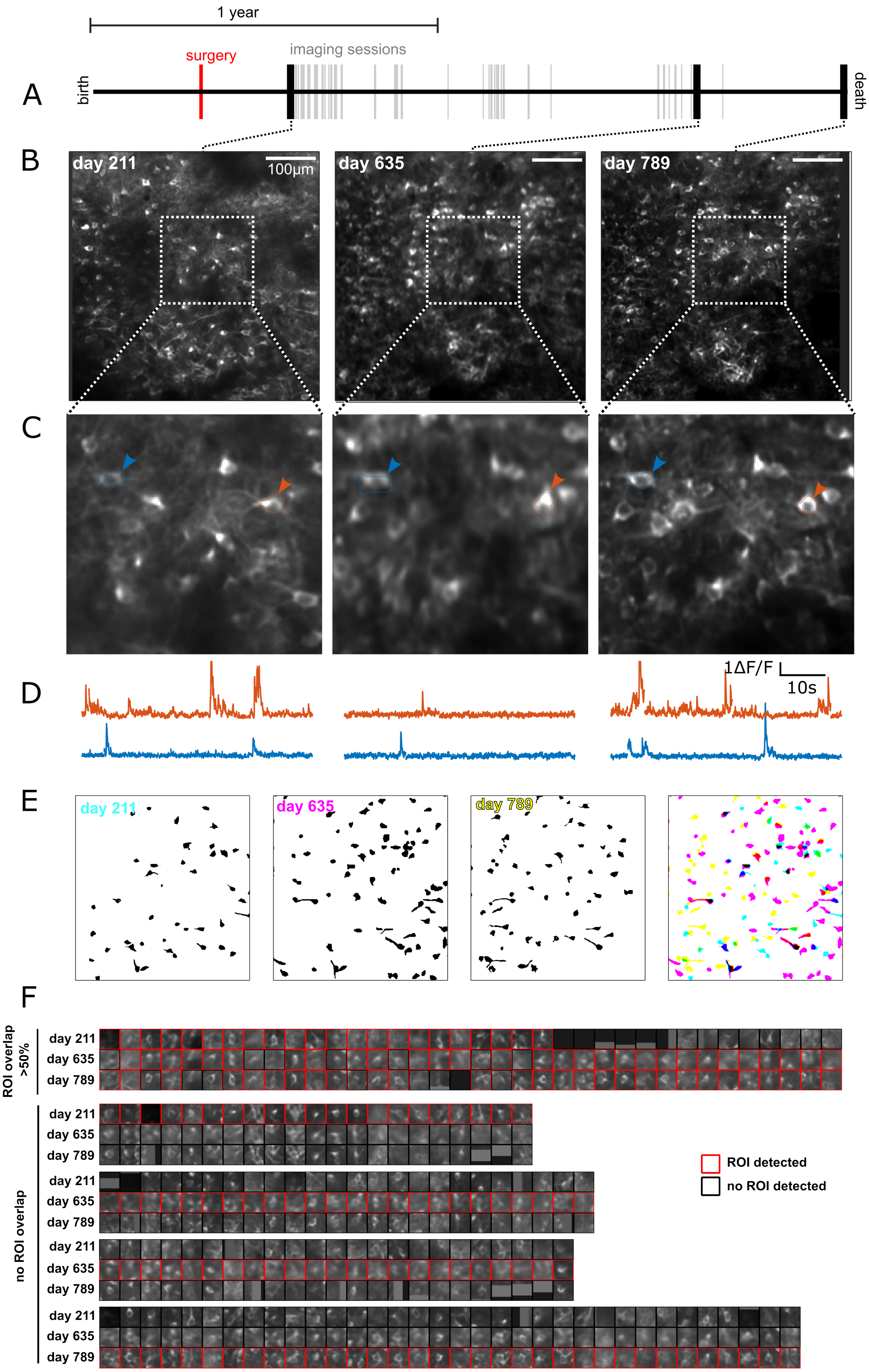

1 year
surgery
imaging sessions
A
birth
death
B
day 211
100µm
day 635
day 789
C
D
1ΔF/F
10s
E
day 211
day 635
day 789
F
ROI overlap >50%
day 211
day 635
day 789
no ROI overlap
ROI detected
no ROI detected

Figure S7 – Same cells are visible and show activity over 19 months

Different field of view for the same animal shown in Fig. 6. **A –** Life line shows the extent of the animal's total life, 25 months. Awake behaving, magnetic head-fixation imaging sessions are shown as ticks. Bold ticks are example sessions illustrated below. **B** Average fluorescence fields of view for three example sessions. **C** Zoomed in details of the fields of view. Two corresponding cells are marked. **D** Calcium fluorescence traces for the two cells indicated above. Gaps in the calcium trace reflect the concatenation of individual trials. **E** The ROI masks for each of the three illustrated sessions in black. With the overlap shown in the fourth panel. The different color combinations correspond to the overlap of the detected ROIs; blue (1,1,0), red (0,1,1), green (1,0,1) Black areas correspond to cells detected in all three FOVs (1,1,1). **F** Zoomed in image patches for all the detected ROIs in the aligned FOVs. Red boarders show the patch of a detected ROI in a session, the black boarders show a corresponding patch in the aligned FOV when no ROI was detected (by our activity-based algorithm). The first row corresponds to cells that are detected and share >50% ROI area overlaps in at least two sessions. Remaining rows correspond to cells that are detected only in a single session. Some patches are outside the boarder of the aligned FOV in some sessions (shown as a black or sharp grey boarder).

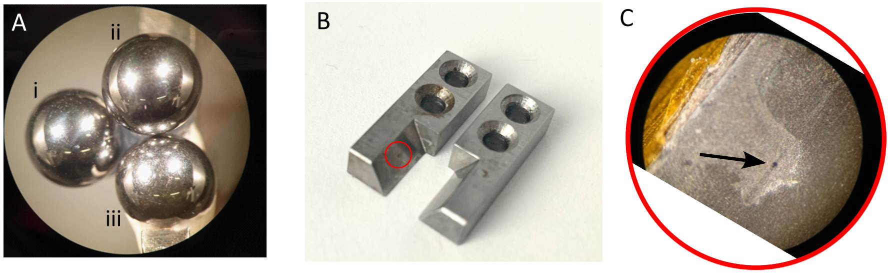

Figure S8 – Bearing surfaces are subject to wear.
In cases where electrical continuity detection became unreliable or intermittent, replacement of bearing surfaces resolved the issue. **A –** showing three 1/4" diameter bearing balls under magnification. I and ii are new bearing balls, and iii has been in use, attached to a head-plate carried be an animal for many months, and shows signs of wear evidences by the higher number of surface scratches. **B** The steel slot bearing pieces also showed signs of wear and brinelling (denting) over extended use. **C** Shows a microscope image of the contact point highlighted in B, with the arrow pointing to the worn contact point.
