# Supplementary material for: Magnetic voluntary head-fixation in transgenic rats enables lifespan imaging of hippocampal neurons
Source: Nat Commun. 2024 May 16;15:4154. doi: 10.1038/s41467-024-48505-9 (PMC11099169; doi:10.1038/s41467-024-48505-9)
Supplement: Supplementary file 3 — Description of Additional Supplementary Files [file 41467_2024_48505_MOESM3_ESM.pdf]

<u>Description of Additional Supplementary Files</u>

Supplementary Movie Legends

Supplementary Movie 1: High magnification field of view for three trials (three columns) in the long fixation experiment. The top element is the raw calcium fluorescence movie, and below that is the same video but motion corrected.

Supplementary Movie 2: Showing head-fixation and imaging during two trials of the odor guided navigation task. The animal head fixes (upper left panel) and imaging starts, scattered infrared light is visible from the un-shuttered imaging laser. During imaging the raw calcium imaging movie of a wide view of CA1 pyramidal cells is shown on the right. The colored square indicates the odor delivery duration. Calcium transients are visible in some cells. After the fixation period has been completed, choice doors open to the left and right, giving the animal access to the rest of the maze. The animal then runs to the correct remote location (bottom left panel) to collect the reward.
