# Supplementary material for: Magnetic voluntary head-fixation in transgenic rats enables lifespan imaging of hippocampal neurons
Source: Nat Commun. 2024 May 16;15:4154. doi: 10.1038/s41467-024-48505-9 (PMC11099169; doi:10.1038/s41467-024-48505-9)
Supplement: Supplementary file 6 — Reporting Summary [file 41467_2024_48505_MOESM6_ESM.pdf]

nature portfolio

Corresponding author(s): P D Rich, D W Tank

Last updated by author(s): Feb 12, 2024

Reporting Summary

Nature Portfolio wishes to improve the reproducibility of the work that we publish. This form provides structure for consistency and transparency in reporting. For further information on Nature Portfolio policies, see our Editorial Policies and the Editorial Policy Checklist.

Statistics

For all statistical analyses, confirm that the following items are present in the figure legend, table legend, main text, or Methods section.

n/a	Confirmed	
☐	☒	The exact sample size (*n*) for each experimental group/condition, given as a discrete number and unit of measurement
☒	☐	A statement on whether measurements were taken from distinct samples or whether the same sample was measured repeatedly
☒	☐	The statistical test(s) used AND whether they are one- or two-sided<br>*Only common tests should be described solely by name; describe more complex techniques in the Methods section.*
☒	☐	A description of all covariates tested
☒	☐	A description of any assumptions or corrections, such as tests of normality and adjustment for multiple comparisons
☐	☒	A full description of the statistical parameters including central tendency (e.g. means) or other basic estimates (e.g. regression coefficient) AND variation (e.g. standard deviation) or associated estimates of uncertainty (e.g. confidence intervals)
☒	☐	For null hypothesis testing, the test statistic (e.g. *F*, *t*, *r*) with confidence intervals, effect sizes, degrees of freedom and *P* value noted<br>*Give P values as exact values whenever suitable.*
☒	☐	For Bayesian analysis, information on the choice of priors and Markov chain Monte Carlo settings
☒	☐	For hierarchical and complex designs, identification of the appropriate level for tests and full reporting of outcomes
☒	☐	Estimates of effect sizes (e.g. Cohen's *d*, Pearson's *r*), indicating how they were calculated

Our web collection on statistics for biologists contains articles on many of the points above.

Software and code

Policy information about availability of computer code

Data collection	Software for the voluntary magnetic head-fixation system are available at the following address. https://github.com/dylan2106/Magnetic-Voluntary-Head-fixation.
Data analysis	Software for the ray tracing experiments is available at the following address. https://github.com/dylan2106/two_photon_collection_cannula

For manuscripts utilizing custom algorithms or software that are central to the research but not yet described in published literature, software must be made available to editors and reviewers. We strongly encourage code deposition in a community repository (e.g. GitHub). See the Nature Portfolio guidelines for submitting code & software for further information.

Data

Policy information about availability of data

All manuscripts must include a data availability statement. This statement should provide the following information, where applicable:

- Accession codes, unique identifiers, or web links for publicly available datasets
- A description of any restrictions on data availability
- For clinical datasets or third party data, please ensure that the statement adheres to our policy

Full schematics and , design files for the voluntary magnetic head-fixation system are available at the following address. https://github.com/dylan2106/Magnetic-Voluntary-Head-fixation.

The source data generated in this study have been deposited in the Zendo database 10.5281/zenodo.10651825, and are provided with this paper.
Thy1-Gcamp6f- 8 rats are available from the Rat Resource & Research Center (www.rrrc.us submission #1010).

Research involving human participants, their data, or biological material

Policy information about studies with human participants or human data. See also policy information about sex, gender (identity/presentation), and sexual orientation and race, ethnicity and racism.

Reporting on sex and gender	N/A
Reporting on race, ethnicity, or other socially relevant groupings	N/A
Population characteristics	N/A
Recruitment	N/A
Ethics oversight	N/A

Note that full information on the approval of the study protocol must also be provided in the manuscript.

Field-specific reporting

Please select the one below that is the best fit for your research. If you are not sure, read the appropriate sections before making your selection.

☒ Life sciences ☐ Behavioural & social sciences ☐ Ecological, evolutionary & environmental sciences

For a reference copy of the document with all sections, see nature.com/documents/nr-reporting-summary-flat.pdf

Life sciences study design

All studies must disclose on these points even when the disclosure is negative.

Sample size	Sample sizes were chosen to allow for sufficient demonstration of the techniques presented, 9 animals. 9/9 for successful training gives the 99% lower CI of true success rate at 92% (matlab - [phat,pci] = binofit(9,9,0.99)); providing a high confidence that most animals are able to be trained.
Data exclusions	Only neurons with at least one calcium transient are able to be identified by constrained non-negative matrix factorization (CNMF). Therefore, cells in the hippocampus that were silent for the entire imaging session were not included.
Replication	All of the 9 animals that were trained on the task were able to successfully reach the criteria for successful vountary head-fixation. 7/9 animals were used for long term imaging experiments, with number of session for each animal = 60,27,62,38,44,7,23
Randomization	There was only one experimental group, so no randomization was needed.
Blinding	There was only one group, so no group allocation blinding was needed

Reporting for specific materials, systems and methods

We require information from authors about some types of materials, experimental systems and methods used in many studies. Here, indicate whether each material, system or method listed is relevant to your study. If you are not sure if a list item applies to your research, read the appropriate section before selecting a response.

Materials & experimental systems

n/a	Involved in the study	
☒	☐	Antibodies
☒	☐	Eukaryotic cell lines
☒	☐	Palaeontology and archaeology
☐	☒	Animals and other organisms
☒	☐	Clinical data
☒	☐	Dual use research of concern
☒	☐	Plants

Methods

n/a	Involved in the study	
☒	☐	ChIP-seq
☒	☐	Flow cytometry
☒	☐	MRI-based neuroimaging

Animals and other research organisms

Policy information about studies involving animals; ARRIVE guidelines recommended for reporting animal research, and Sex and Gender in Research

Laboratory animals	9 male and female transgeneic rats (Long evans background) were used, ages ranges from 3 - 25 months
Wild animals	no wild animals were used
Reporting on sex	5 male and 4 female animals were used in this study. No sex based analysis was performed because all individuals both sexes were able to be trained to criteria in the voluntary head-fixation.
Field-collected samples	no field-collected samples were used
Ethics oversight	The Princeton IACUC Committee provided ethical oversite for the study protocol under protocol #1837

Note that full information on the approval of the study protocol must also be provided in the manuscript.
